# G2/M-Phase Checkpoint Adaptation and Micronuclei Formation as Mechanisms That Contribute to Genomic Instability in Human Cells

**DOI:** 10.3390/ijms18112344

**Published:** 2017-11-06

**Authors:** Danî Kalsbeek, Roy M. Golsteyn

**Affiliations:** Cancer Cell Laboratory, Department of Biological Sciences, University of Lethbridge, Lethbridge, AB T1K 3M4, Canada; dani.kalsbeek@uleth.ca

**Keywords:** checkpoint adaptation, checkpoint kinase 1 (Chk1), chromothripsis, cyclin-dependent kinase 1 (Cdk1), human colon adenocarcinoma (HT-29) cells, micronuclei

## Abstract

One of the most common characteristics of cancer cells is genomic instability. Recent research has revealed that G2/M-phase checkpoint adaptation—entering mitosis with damaged DNA—contributes to genomic changes in experimental models. When cancer cells are treated with pharmacological concentrations of genotoxic agents, they undergo checkpoint adaptation; however, a small number of cells are able to survive and accumulate micronuclei. These micronuclei harbour damaged DNA, and are able to replicate and reincorporate their DNA into the main nucleus. Micronuclei are susceptible to chromothripsis, which is a phenomenon characterised by extensively rearranged chromosomes that reassemble from pulverized chromosomes in one cellular event. These processes contribute to genomic instability in cancer cells that survive a genotoxic anti-cancer treatment. This review provides insight into checkpoint adaptation and its connection to micronuclei and possibly chromothripsis. Knowledge about these mechanisms is needed to improve the poor cancer treatment outcomes that result from genomic instability.

## 1. Introduction

Most cancer cells have genomes that are extensively altered relative to normal, non-transformed human cells. The Cancer Genome Sequencing Atlas project has sequenced the genomes of solid tumours from thousands of patients, and all showed different chromosomal organisation and DNA sequences. This genomic complexity likely contributes to treatment inefficiency [1,2,3].

One way to target cancer cells in treatments is to induce irreparable amounts of DNA damage by the application of genotoxic agents. These agents activate the cell cycle checkpoints, leading to cell cycle arrest and/or cell death [4]. However, research has shown that some cancer cells treated with genotoxic anti-cancer drugs can overcome a G2 phase cell cycle arrest and enter mitosis with damaged DNA, a mechanism called G2/M-phase checkpoint adaptation [5,6,7,8]. Most cells that undergo G2/M-phase checkpoint adaptation (hereafter called checkpoint adaptation) after treatment with a genotoxic agent die in mitosis. However, a small number of those cells survive, and are likely to have extensively altered genomes due to the processes of repair and adaptation [6,8]. Recent research on checkpoint adaptation led to the idea that this process contributes to genomic instability, hence genomic complexity [9].

Investigation of checkpoint adaptation led to the finding that it causes the formation of micronuclei in cells [10,11]. Micronuclei are distinct from the main nucleus and encapsulate full lagging chromosomes or fragments of chromosomes that are not incorporated in the main nucleus after anaphase [12,13,14]. Micronuclei contribute to genomic instability, and are a characteristic of cancer cells. Cells that undergo checkpoint adaptation show a 50% increase in the number of micronuclei compared with non-treated cancer cells [10,11]. The cells containing micronuclei are able to survive for several cycles, and chromosomes enclosed in these micronuclei could be reunited in the main nucleus of the daughter cells. Furthermore, more than 7.5% of micronuclei in cancer cells were found to contain shattered chromosomes, which is part of a phenomenon called chromothripsis [14,15,16,17,18]. Micronuclei are proposed to play a role in the initiation of this phenomenon [14,19,20].

This review provides insight into checkpoint adaptation and its connection to micronuclei and chromothripsis. In reviewing these topics, we hope to provide knowledge for a better understanding of mechanisms that might be involved in poor cancer treatment outcomes as a result of genomic instability. Altered genomes can result in cells that do not respond to death signals, and hence become treatment resistant [21]. We suggest that cells treated with DNA damaging anti-cancer agents and survive checkpoint adaptation become candidates for cells that sustain further genomic change through the formation of micronuclei and chromothripsis.

## 2. Checkpoint Adaptation

To maintain the integrity of the genome, cells have cell cycle checkpoints that detect damaged DNA and aneuploidy, and prevent them from transmitting changed genomes to daughter cells [22,23]. The majority of cancer cells are mutated in genes encoding tumour suppressors such as p21, p53, or retinoblastoma [3,24]. In normal cells, the activation of p53 leads to a G1/S-checkpoint arrest that prevents cells from proliferating. Mutation in p53 results in a defective G1/S-checkpoint, which means that cells can only rely on a G2/M-checkpoint to detect damaged DNA [22]. It is noteworthy that the absence of p53 may be an obligatory event for cells to tolerate a changed genome [25].

In addition to the p53-dependent checkpoint, cells respond to damaged DNA by the DNA damage ataxia telangiectasia mutated (ATM)/ataxia telangiectasia and Rad3-related protein (ATR) signaling in which histone H2AX is phosphorylated (histone γH2AX) [26]. This pathway also phosphorylates checkpoint kinase 1 (Chk1), which in turn prevents the activation of cyclin-dependent kinase 1 (Cdk1) by the induction of Wee1 kinase activity and the inactivation of cell division cycle 25 (Cdc25) phosphatases [27,28,29]. Cdk1 is a protein kinase that phosphorylates a large number of substrates whose activities are required for mitosis. Activation of Chk1 thus arrests cells in the G2/M-checkpoint [11], and its inactivation by either dephosphorylation [6,30] or degradation [31] is required before cells enter mitosis under conditions of damaged DNA signaling. Whereas p53 is mutated in the majority of human tumours, Chk1 mutations in tumours are extremely rare [32]. Chk1 can be activated by nearly all genotoxic treatments [7], including neo-synthetic compounds [33]. The activation of the DNA damage checkpoints by UV irradiation appears to be more dependent upon the p53 pathway than the Chk1 pathway [34].

It has been observed that after treatment with genotoxic agents such as irradiation, topoisomerase I inhibitors, topoisomerase II inhibitors, or cross-linking agents such as cisplatin, cancer cells are able to enter mitosis with damaged DNA. This process is called checkpoint adaptation, and is characterised by three steps: initiation of cell cycle arrest at the G2/M-checkpoint by damaged DNA, overcoming the arrest, and entering mitosis with unrepaired DNA damage [30,33,35,36,37,38]. To undergo checkpoint adaptation, Chk1 is either degraded [31] or dephosphorylated [6,30], which enables Cdk1 to be activated despite the presence of damaged DNA [11]. Checkpoint adaptation is likely to be a primary pathway that leads to cell death after treatment with pharmacological amounts of genotoxic agents in cancer cells [6,39].

### Identification of Checkpoint Adaptation

Checkpoint adaptation was first described by Sandell et al. in 1993 as a process in the yeast *Saccharomyces cerevisiae* that allows cells to enter mitosis with unrepaired DNA [40]. They demonstrated that specifically mutated haploid yeast cells, which were unable to repair double-stranded DNA breaks (DSB), arrested at the G2/M-checkpoint after the induction of DNA damage. However, instead of undergoing cell death, these cells overcame the checkpoint and entered mitosis with damaged DNA [40]. Further studies in yeast revealed that cells identified a relationship between single-stranded DNA and the capacity to exit the G2/M-phase checkpoint [41]. Later, pharmacological approaches enabled the detection of human cancer cells that entered mitosis with damaged DNA [42]. Furthermore, human cancer cells treated by ionizing radiation (IR) underwent several cell cycles. The DNA in these cells contained damage in the form of gaps, acentric DNA fragments, and chromatid breaks [38]. It was suggested that human cancer cells with damaged DNA induced by IR can overcome the G2/M-checkpoint and enter mitosis despite the genomic alterations [5]. Syljuåsen investigated this hypothesis in 2006, and found that human osteosarcoma cells treated with 6 Gy IR arrested at the G2/M-transition, where after they entered mitosis without repairing the DNA. This complies with the characteristics of checkpoint adaptation [30]. In cases where the damage to DNA can be repaired, cells undergo a process known as checkpoint recovery, and continue to proliferate [23].

**Figure 1:**
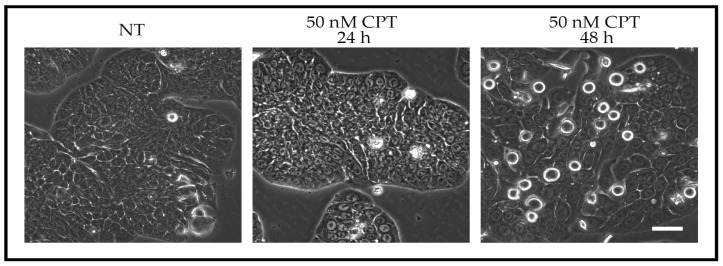
Checkpoint adaptation in human cancer cells. Human HT-29 cells were either not treated (NT), or treated with a cytotoxic amount of camptothecin (50 nM CPT) and observed by phase contrast microscopy at 24 or 48 h. The rounded cells in the 48 h image are in mitosis as they undergo the G2/M-phase checkpoint adaptation. Refer to Kubara et al. [6] or Swift and Golsteyn [8] for additional experimental data of damaged DNA in rounded, mitotic cells. Bar represents 50 µm.

More recent work investigating checkpoint adaptation in human cancer cells suggests that mitosis plays an important role in the response to treatment with genotoxic agents [6]. Cell-based assays were used to observe the events that follow the arrest at the G2/M-transition after cells were treated with genotoxic agents that induced DNA damage. Treatment with cytotoxic and pharmacological amounts of camptothecin (25 nM CPT) induced cells to acquire a rounded morphology after 40 or more hours of treatment, and tested positive for mitotic markers (Figure 1). Human colon adenocarcinoma (HT-29) cells treated with a pharmacological concentration of CPT showed histone γH2AX foci in all of the cells arrested at the G2/M-transition with activated Chk1 [6]. In addition to CPT, cisplatin-treated or etoposide-treated cells also showed the ability to undergo checkpoint adaptation [7,8]. In cells treated with 30 µM of cisplatin, a pharmacological concentration, 80% of treated cells entered mitosis before dying. On the other hand, at a supra-pharmacological cytotoxic concentration of 100 µM, only 7% of treated cells entered mitosis, whereas the remaining cells died by apoptosis. These outcomes led to the prediction that checkpoint adaptation is a key pathway in cell death induced by genotoxic agents [8]; however, checkpoint adaptation in human cells treated by ultraviolet light energy has not yet been reported. Notably, approximately 2% of cells that underwent checkpoint adaptation survived and showed increased numbers of micronuclei [6,11]. For an additional discussion on the importance of concentrations of cytotoxic compounds and cell death, see Swift and Golsteyn [7], and Brown and Attardi [39].

## 3. Micronuclei

Micronuclei are small DNA containing structures surrounded by one lipid bilayer, which are independent from the main nucleus in a cell. Whole chromosomes, as well as chromosome fragments, can be in micronuclei, depending on how it was formed. The DNA in micronuclei can be replicated, transcribed, and repaired in a manner that is similar to the main nucleus [43]. However, research on micronuclei in vitro has shown that they may have defects in the localisation of structural proteins, such as pores, when compared with the main nucleus [14,44,45,46,47]. Although micronuclei are considered as a marker for genomic change, there are biological processes in which micronuclei appear normally, such as during the embryonic development of species such as *Xenopus* [48]. The number and size of micronuclei can vary in the cell depending on the origin of the micronucleus [13,49,50]. Micronuclei are tightly associated with mitotic errors, and therefore they are considered indicators of aneuploidy and genomic instability [51].

### 3.1. Factors Leading to the Formation of Micronuclei

The formation of micronuclei can be initiated by errors in chromosome segregation or damaged DNA, which can be induced by chemical and physical factors. The factors are divided into two groups: aneugens and clastogens. Aneugens induce the formation of micronuclei that contain complete chromosomes by targeting the segregation of chromosomes into a nucleus. By contrast, clastogens result in micronuclei that contain acentric chromosome fragments, which are caused by the induction of DNA breaks [19,43,52].

#### 3.1.1. Aneugens

Aneugens are chemical antimitotic agents that affect mitotic spindle formation, and thus the segregation of chromosomes. Cells treated with such agents can form micronuclei that contain intact chromosomes. These chemicals can arrest cells in the cell cycle and cause death, including by apoptosis activated by a p53-dependent pathway [43]. Human breast carcinoma cells treated with taxol, vincristine, or nocodazole showed a 20% increase in micronuclei number and cell death rate [53]. Other types of aneugens can suppress DNA and histone methylation, and disrupt the condensation of chromosomes in the centromere [19,54]. These agents can also show a clastogenic effect by the induction of DSBs. Furthermore, metals that bind to DNA and proteins show both aneugenic and clastogenic effects. They can influence gene expression, the condensation of DNA, and mitotic spindle assembly [43,55].

#### 3.1.2. Clastogens

Agents that display a clastogenic effect are anthracycline agents that perturb DNA replication and repair [43,55]. In addition, hypoxia and oxidative stress have also been shown to contribute to the formation of micronuclei in cells [56,57]. Platinum-based drugs, such as cisplatin, which are used in testicular and ovarian cancers [4,58], form mono-functional DNA adducts that block replication, which leads to DNA strand breaks and thus causes a clastogenic effect [7]. Treatment with cisplatin was shown to increase the number of micronuclei by 24% to 48% in cultured human glioblastoma cells that underwent checkpoint adaptation [11]. Most of the physical factors are clastogenic. Physical factors that can lead to micronuclei formation in cells include, for example, changes in pressure and temperature, radiation, UV, and ultrasound [43]. Radiation exposure causes various DSBs that can lead to chromosomal rearrangements when the fragments are fused in a random order [16,59]. Exposure to 2 Gy of radiation resulted in DSBs in 80% of human fibroblast cells, and 48 h after treatment, 80% of the cells contained micronuclei, which correlates with the fraction of cells with DSBs [45].

### 3.2. Micronuclei Formation Pathways

The structure of a micronucleus is dependent on how it was formed. Chemical and physical factors are attributable to the different mechanisms through which micronuclei can be formed. These mechanisms involve changes in protein quantity or defects caused by mutation that affect the nuclear envelope, the structure of the chromosome centromeric region, attachment to spindle microtubules, DSBs, chromoanagenesis, oncogene amplification, and double minute (DM) chromosomes [43]. In the case of the induction of micronuclei by checkpoint adaptation, disruptions in the structure of centromeric region, defects of attaching to spindle microtubules, and DSBs are believed to be involved.

#### 3.2.1. Defective Microtubule–Kinetochore Attachments

Most micronuclei are formed at the end of mitosis. Defects in the separation of chromosomes in the daughter nucleus leads to the occurrence of aneuploid cells. These cells are used as markers for tumours, and may play a predominant role in tumour initiation and development. Defects in chromosome segregation during mitosis can change how the genome is organised in a cell [13,55]. For example, it may lead to daughter cells that contain micronuclei that enclose either whole intact chromosomes, or chromosomes with structural aberrations [60].

Irregularities in the centromeric region structure of chromosomes and kinetochores can be caused by centromeric DNA replication defects, DNA and histone methylation defects in the centromeric region, and mutations in genes that encode for kinetochore proteins [13,53,61,62,63]. These defects can lead to the formation of micronuclei, as these chromosomes cannot properly align at the metaphase plate [14]. Delayed chromosomes are randomly distributed to one of the daughter cells at the end of mitosis, where they form a micronucleus. These micronuclei may have different fates, depending upon the nuclear envelope that forms around them. If the lagging chromosome is enclosed by a normal organised nuclear envelope, it might escape subsequent DNA damage. Under these conditions, if a chromosome was unable to bind to microtubules because of damage in the kinetochore, the damage can be repaired, and the chromosome might attach to the mitotic spindle in the subsequent mitosis and no longer form a micronucleus. By contrast, if the chromosome is enclosed by a nuclear envelope that contains irregularities in its organisation, it might be not be correctly replicated, transcribed, or repaired, leading to additional changes in the genome [14,43].

Normal kinetochores and microtubules can form incorrect interactions during mitosis, which can be detected and resolved by checkpoint mechanisms. When these incorrect interactions are not resolved, such as when both kinetochores attach to the same division pole, then both chromosomes will be directed to the same daughter cell. When microtubules from both division poles bind to the same kinetochore, a merotelic chromosome interaction is formed, which can also lead to micronuclei harbouring intact chromosomes [64,65,66]. Combinations of microtubule dynamics that are guided by motor proteins [62] and protein kinases such as Aurora B ensure correct kinetochore–microtubule interactions [67]. Aurora B regulates these events by phosphorylating substrates that are proximal to it, depending upon mechanical tension. The overexpression of proteins in the Aurora B mediated correction mechanism can lead to the hyperstabilisation of kinetochore–microtubule attachments, which increases the number of incorrectly attached kinetochores [68]. Furthermore, new substrates for Aurora B have been identified, such as 53BP1, which participates in kinetochore and microtubule interactions, and if mutated, may lead to micronuclei [69].

#### 3.2.2. DSBs and DM-Chromosomes

Acentric fragments are formed during mitosis as a result of DSBs and DM chromosomes, which is a clastogenic effect. These fragments are unable to interact with microtubules, and are randomly divided between daughter cells. During telophase, these fragments are encapsulated by a generated nuclear envelope that contains lamina and nuclear pores. These micronuclei can be functionally active, and are not excluded from the cell [70,71]. One characteristic of these micronuclei is breaks in their DNA. These lesions are caused by the incomplete replication of the DNA in the preceding interphase. Micronuclei that are formed during this process are likely to be degraded. However, when there are no DNA breaks in micronuclei, and the nuclear envelope develops normally, micronuclei are able to function normally in the following interphase [43].

#### 3.2.3. Micronuclei Formation in Interphase

Micronuclei can also be formed during interphase by a mechanism called nuclear blebbing. Extrachromosomal pieces of the main nucleus are transported to the nuclear envelope, where a bud is formed. Separation from the main nucleus forms a micronucleus. These micronuclei are typically small, and are located in proximity of the main nucleus. There are two different disturbances that are described as leading to this type of micronuclei: amplified oncogenes, and the appearance of multiple DSBs [43].

One of these disturbances leads to the formation of the double minute (DM) micronuclei. DM chromosomes are small, circular fragments of DNA that occur extrachromosomally, and play an important role in tumour genetics. These DM chromosomes mostly contain genes that are amplified. These DM chromosomes are formed in response to the structural rearrangements of chromosomes, such as chromothripsis, after which the pieces are joined together in a circular matter [15,16]. Most DM chromosomes have increased copies of oncogenes, which contributes to the proliferative activity of tumour cells [15]. The DM micronucleus emerges as a result of the occurrence of these DM chromosomes. It is suggested that the occurrence of DM micronuclei can be a mechanism to exclude DM chromosomes from the cell [72,73]. DM chromosomes occur at the periphery of the nucleus during interphase, where they can pass through a lamina break, and get included into a nuclear bud. This bud is a precursor for micronucleus formation, including the DM chromosomes. This suggests that these DM micronuclei that are formed during interphase have irregular nuclear envelopes that restrict them in their functional activity [74]. These micronuclei can be degraded in the cell [12] or extruded out of the cell [72].

Nuclear blebbing also produces another type of micronuclei in interphase cells. These micronuclei enclose DNA fragments that are damaged. Treatment with radiation results in many DSBs. The damaged DNA is positive for histone γH2AX, and 24 h after treatment, many micronuclei test positive for γH2AX [75]. This can be seen as indirect proof that these micronuclei are formed as a result of nuclear blebbing [43]. It is suggested that enclosing damaged DNA fragments in micronuclei is associated with failing DNA repair mechanisms and cell cycle checkpoints. When this fails, the damaged DNA is included into a micronucleus, and subsequently degraded by autophagy [12].

### 3.3. Nuclear Envelope

The nuclear envelope is formed from an association of endoplasmic reticulum membranes with chromatin followed by fusion of the endoplasmic reticulum sheets in late anaphase and telophase [76,77]. This formation requires the recruitment of proteins, which eventually form the major structures of the envelope such as nuclear pore complexes, nuclear lamina for envelope stabilisation, and the proteins for the inner nuclear membrane [78]. The inner nuclear membrane proteins bring the endoplasmic reticulum to the chromatin, and connect the lamina to the membrane [79]. The nuclear envelope is crucial to chromatin organisation in the main nucleus, as it determines the compactness, functional activity and structural organisation of chromatin. These factors are influenced by proteins that are involved in the structure maintenance, and the transport between the nucleus and the cytoplasm [43].

Terradas et al. reported that the capacity to replicate DNA differed among micronuclei in cells. Almost half of the micronuclei that were formed in response to irradiation in human lymphocyte cells were able to replicate their DNA. However, only 9% of the micronuclei in human fibroblast cells had this capacity after exposure to radiation [45,80]. DNA replication in micronuclei can occur asynchronously relative to the replication of the main nuclear DNA. Delayed replication is thought to be caused by a reduction in the number of nuclear pores and deficiencies in the transport of proteins between the micronucleus and the cytoplasm. The integrity of the nuclear envelope is necessary to ensure the access of macromolecules to the genome inside the envelope.

Undamaged micronuclei may also show dysfunctions in the recruitment of proteins involved in DNA replication and repair [14,47]. Micronuclei show similarities with the main nucleus; however, the nuclear lamina is not properly organised [81]. As a consequence, the nuclear envelope collapses, and functioning of the undamaged micronuclei are affected, because the changes in lamin B1 organisation impair transcription and replication in the main nucleus [82,83,84]. Hatch et al. identified that the disruption of the micronucleus causes an accumulation of damaged DNA [81]. It was suggested that damaged DNA in the micronucleus is an outcome of a deficient replication event [14,46]. DNA replication sensitises an intact micronucleus to DNA damage that is triggered by micronuclear disruption. The deficiencies in transcription and genomic replication, as well as damaged DNA, can all contribute to aneuploidy in different ways. Defective transcription can lead to a short-term aneuploidy in interphase cells. If segregated chromatin encodes crucial regulators for mechanisms involved in genomic stability, it could lead to permanent genome alterations. Furthermore, deficiencies in replication can lead to the entry into mitosis, with irregular amounts of DNA causing the daughter cells to be aneuploid [14,45,81]. Damaged DNA in micronuclei might lead to DNA shattering in interphase and mitosis. DNA fragments are believed to be reassembled randomly, leading to massive genomic rearrangements, through a process called chromothripsis [14,15]. It is shown that there is a clear association between the collapse of the nuclear envelope in the micronucleus, and an increase in the amount of DNA damage [81]. This will lead to the pulverisation of the immature condensed chromosomes when cells enter mitosis. This pulverisation is accompanied by several DSBs and chromothripsis [14].

The formation of one type of micronuclei can lead to the production of other types of micronuclei in the subsequent cell cycle. In this case, the newly formed micronucleus is likely to contain a nuclear envelope with fewer than normal nuclear pores, which leads to the deregulation of the nuclear-cytoplasmic transport. These micronuclei will undergo delayed genomic replication compared with the main nucleus, and will continue through the G2-phase [14]. However, these micronuclei are likely to constitute the basis for the formation of premature condensed chromosomes. Mitotic entrance with DNA that is not completely replicated results in the development of various micronuclear DSBs or chromothripsis, which can lead to amplification of the genome and formation of DM micronuclei [14,85].

**Figure 2:**
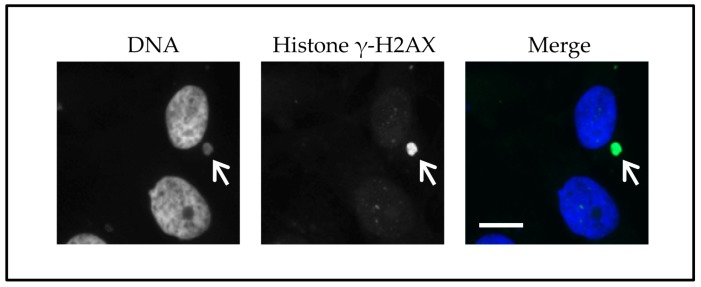
Micronuclei can signal damaged DNA independently of a main nucleus. HT-29 cells that survived checkpoint adaptation were cultivated. Cells were then fixed, stained with DAPI (blue) to identify nuclei and micronuclei, and treated with antibodies to histone γ-H2AX (green) to detect damaged DNA. The arrow points to a micronucleus that is positive for damaged DNA, whereas the main nuclei do not signal damaged DNA. Bar equals 5 μm. For further information, see Lewis and Golsteyn [11].

The asynchronous replication observed in micronuclei is likely due to a disrupted nuclear envelope that affects the intake of proteins required for DNA replication [18,81]. Another factor that may delay DNA replication is damaged DNA, which could potentially be amplified if a cell enters mitosis. In experiments using cultured M059K cells, almost 50% of the micronuclei contained histone γH2AX, which suggests that these micronuclei had damaged DNA (Figure 2) [11]. The damage signal was linked to DNA replication, because the frequency of histone γH2AX positive micronuclei was reduced when DNA replication was blocked. An increase in histone γH2AX positive micronuclei could be induced by treating cells with cisplatin. This suggests that the asynchronously DNA replication of the main nucleus and the micronucleus may be the cause of continuous DNA damage.

Micronuclei in normal cells, and in some cancer cells, can result in a p53-mediated cell cycle arrest followed by apoptotic cell death [53,86]. However, there is evidence that cancer cells have the ability to exclude micronuclei [13,55], which can promote survival when cells are treated with chemotherapy [43]. In p53 wildtype cancer cells, the exclusion of the micronucleus can result in the stimulation of the cell cycle and tumour development. Genetic alterations drive aneuploidy and genomic instability, which in turn promote the cancer phenotype. Micronuclei are found to be major contributors to genomic instability and genomic rearrangements [43].

## 4. Checkpoint Adaptation and Micronuclei

Chang et al. identified that cells that underwent checkpoint adaptation after treatment with genotoxic agents contained increased levels of micronuclei [87]. This relationship was found in fibrosarcoma cells (HT1080). The genotoxic agents, doxorubicin, aphidicolin, cisplatin, etoposide, vincristine, cytarabine and γ-irradiation, were tested at concentrations that induced 85% growth inhibition after 4 days of continuous exposure. Notably, the treated cells showed a 45–66% increase in the number of micronuclei [87]. Lewis and Golsteyn (2016) demonstrated that glioblastoma cells treated with 30 µM cisplatin underwent checkpoint adaptation and had an increased number of micronuclei per cell, more cells that contained micronuclei, and an increased number of nucleoplasmic bridges. These micronuclei persisted for at least 8 days after treatment [11]. Inhibition of Chk1 allowed more cells to enter mitosis with damaged DNA [88], and led to more cells having micronuclei when compared with cells that were treated with a only a genotoxic agent. Finally, preventing checkpoint adaptation by co-treatment with a Cdk1 inhibitor reduced the number of cells that had micronuclei. These findings suggest that checkpoint adaptation is linked to the presence of micronuclei [11]. The notion that HT-29 cells showed an increased number of micronuclei when deficient for Chk1 supports the previous observation [89]. These micronuclei, which possess a nuclear lamina, were able to replicate their genome independently and asynchronously compared with the main nucleus [11,71].

## 5. Chromothripsis

Stephens et al. first described chromothripsis in 2010 by identifying the event whereby hundreds of rearrangements occur in the genome [15]. It is a one-off catastrophic incident in which hundreds of extensive genomic rearrangements occur to one or few chromosomes after the random reattachment of the pulverised chromosomes. It is seen in about 3% of all cancers [15], especially in brain and bone tumours [20]. In the majority of the cases, chromothripsis will lead to cell death, but it is suggested that cells that survive do so because they have acquired a selection advantage due to their extensively altered genomes. These cells are characterised by three different advantageous genomic modifications: the loss of tumour suppressor genes, the gain of function oncogenes, and/or the formation of fusion genes. Chromothripsis can create mutations that can promote cancer development [21]. Neuroblastoma, several myelomas, melanoma, and acute myeloid leukemia all show poor disease outcomes that are strongly associated with chromothripsis [24,90,91,92].

Crasta et al. studied micronuclei in cancer cells and found that DNA in these micronuclei was damaged during its replication. They also found that 7.6% of micronuclei contained pulverised chromosomes [14]. This pulverisation is part of a phenomena called chromothripsis. Micronuclei are proposed to be one of the main factors that cause chromothripsis [17]. In 2015, Zhang et al. provided a first indication of how chromothripsis can be acquired by using live cell imaging and single cell genome sequencing. They suggested that the enclosure of chromosomes in micronuclei is an important factor that contributes to the acquisition of the DNA lesions seen in chromothripsis [17]. These micronuclei are a result of cell division defects, and it was shown that whole chromosomes encapsulated in micronuclei acquire DNA damage. Subsequently, damaged chromosomes can re-enter daughter nuclei, and possibly integrate mutations into the genome [14]. Chromosomes in micronuclei are under-replicated, and accumulate damaged DNA [14,81]. This under-replication of chromosomes in the micronucleus causes an asymmetry in DNA copy number between the daughter nuclei, what identifies the missegregated chromosome and leads to de novo chromosome missegregation. This notion provides evidence for the suggestion that the rearrangements seen in micronucleated cells after division occur on the chromosome that is encapsulated in the micronucleus. Another piece of evidence is that only the missegregated chromosome accumulated chromosomal rearrangements after the division of the micronucleated cell. This feature has never been observed in normally segregated chromosomes after division in micronucleated cells, as well as control cells. To conclude, rearrangements are correlated with the chromatid that has been identified by the gained haplotype in the micronucleus [17].

This intrachromosomal phenomenon, along with the encapsulation of one or more chromosomes into a micronucleus, affects genomic structure. This can result in translocations between the encapsulated chromosomes. Zhang et al. suggest that the reassembly of DNA fragments in a micronucleus can produce ring structures whose formation might be associated with chromothripsis. These ring structures are seen in a number of human cancers with missegregated chromosome origins [17,93]. The formation of ring structures from fragmented chromosomes is a possible mechanism that is responsible for the production of double minute chromosomes [94], which have been linked to chromothripsis [95].

A consequence of chromothripsis is the loss of chromosomes fragments [15]. However, the precise mechanism is not yet understood. Research by Zhang et al. reported that the chromatid can indeed be shattered, with the distribution of DNA pieces between the daughter nuclei. Loss of DNA fragments was explained by the separation of these fragments into daughter nuclei that did not expand and did not become part of the final cell population [17].

### Mechanisms That Might Lead to Chromothripsis

The mechanism that is proposed to be most plausible for chromosome pulverisation in micronuclei is entrance into mitosis before the DNA in the micronucleus has been completely replicated [96,97,98]. The nuclear envelope of a micronucleus fails to disassemble during mitosis, which is crucial to maintain chromosome fragments. The nuclear envelope can be maintained because mitotic Cdks are unable to enter the micronucleus, nor phosphorylate lamins that are required for the disassembly of the nuclear envelope [14]. If the nuclear envelope of the micronucleus disassembles in mitosis, fragments would enter the cytoplasm and would be lost. This failure to disassemble leads to the segregation of intact nuclei to one of the daughter cells. The micronucleus then keeps these chromosome fragments enclosed in a compartment until the following cell cycle. It is suggested that the persistence of a micronucleus into the second interphase after its formation provides a repair mechanism to ligate the fragments that were formed randomly by incomplete DNA replication, and form rearranged chromosomes [16].

Pulverised and rearranged chromosomes can be reincorporated in the main nucleus to contribute to genomic instability, and thus cancer promotion. Although the fate of the micronucleus is still unknown, it is suggested that these micronuclei are excluded from the cell, degraded, or reincorporated in the main nucleus. The first two options result in the loss of these chromosomes, but the third option gives rise to genomic rearrangement [14,17].

## 6. Pharmacological Inhibition of Checkpoint Adaptation

There is now sufficient information available to test pharmacological approaches that can target adaptation to checkpoints in cancer cells. One of the checkpoints targeted is the spindle assembly checkpoint (SAC). The anaphase-promoting complex (APC) can be inhibited by a small molecule tosyl-L-arginine methyl ester (TAME) that displaces the IR tail of Cdc20 or Cdh1. It is suggested that the prodrug, proTAME, prevents the inactivation of SAC [99]. Another approach that is being investigated is the inhibition of the anaphase-promoting complex/cyclosome (APC/C). APC/C is an ubiquitin ligase that triggers the metaphase–anaphase shift and mitotic exit through the ubiquitin-dependent destruction of proteins such as securin and cyclin B1. The small molecule Apcin binds to Cdc20, and inhibits the APC/C-dependent proteolysis and mitotic exit by disrupting the interaction between the APC/C and Cdc20, which prevents the ubiquitination of D-box-containing substrates [100]. Anti-microtubule cancer drugs (AMCDs) can also be used to prevent mitotic exit with deformed spindles by targeting the Fcp1–Wee1–Cdk1 axis. During mitotic arrest induced by AMCDs, Fcp1 activates Wee1 by dephosphorylation, which in turn lowers the Cdk1 activity. This results in a weakened SAC-dependent mitotic arrest, and thus promotes mitotic exit and survival. The inhibition of Wee1 strengthens the SAC and extends mitosis, hence enhancing AMCD-induced cell death [101].

Another checkpoint that is being targeted is the G2/M-checkpoint. One approach is targeting the DNA damage response (DDR). This response regulates DNA damage repair and signaling to cell cycle checkpoints [102]. The dysregulation of the DDR has been associated with cancer, as it alters the response to DNA, damaging anti-cancer therapies. If a DNA repair pathway fails, its function might be taken over by another DDR pathway, which may be increased, and supports resistance to DNA damaging agents. The ATM and ATR pathways signal DNA damage and have multiple downstream targets. Therefore the inhibition of checkpoints is suggested to sensitise DNA-damaging agents. This can be done with an ATM inhibitor such as KU55933, Wee1, and Cdc25 inhibitors, or Chk1 inhibitor UCN-01. More recently two novel ATR inhibitors, VE-821 and NU6027, have been identified to sensitise cells to various DNA damaging agents [103]. By inhibition of this pathway, the cells bypass the checkpoints, enter mitosis, and undergo mitotic catastrophe and, eventually, cell death [104]. Most of the inhibitors showed little impact on cell cycle distribution or viability. However, they prevented cell cycle arrest and increased the cytotoxicity of DNA damaging agents [103].

## 7. Conclusions

**Figure 3:**
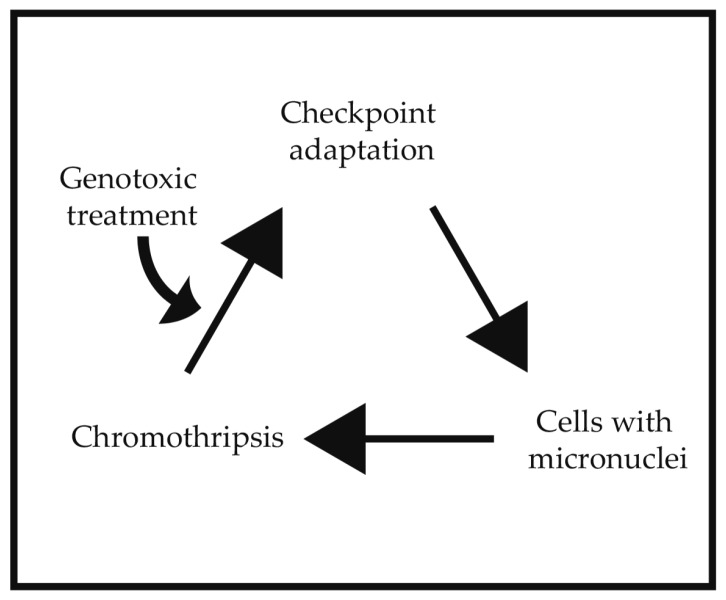
A schematic diagram of a relationship between checkpoint adaptation, micronuclei, and chromothripsis. A genotoxic treatment starts the cycle by initiating checkpoint adaptation.

We are proposing that checkpoint adaptation, micronuclei, and chromothripsis are distinct cancer cell phenomena that are linked by biological steps and enhance genomic instability (Figure 3). In experimental models, checkpoint adaptation provides an occasion for cells to produce micronuclei. The micronuclei isolate a portion of the genome from the main nucleus and create conditions that could lead to chromothripsis. Cells have checkpoints and DNA repair mechanisms as well as death pathways that greatly reduce the likelihood of genomic instability; however, examples of these events have been observed in experimental models. Checkpoint adaptation is a more frequent outcome than apoptosis when pharmacological concentrations of genotoxic agents are used in experimental models [8]. Although most of these cells die in mitosis, a small number of cells survive and generate micronuclei that are prone to DNA damage and chromothripsis. All of the aforementioned processes may contribute to genomic instability, and thus are able to promote the development of resistance against treatments. As a new expanding field, we require a better understanding of the biochemical pathways that participate in these events. In the case of checkpoint adaptation, the role of mitotic kinases such as Cdk1 may be re-evaluated with a view on genomic instability in cancer cells. A better understanding of the acquisition of genomic instability in cancer cells will provide more insight in how cancer patients can be treated more effectively.

## References

[1] The Cancer Genome Atlas Network (2008). Comprehensive genomic characterization defines human glioblastoma genes and core pathways. Nature.

[2] The Cancer Genome Atlas Network (2012). Comprehensive molecular characterization of human colon and rectal cancer. Nature.

[3] Kandoth C., McLellan M.D., Vandin F., Ye K., Niu B., Lu C., Xie M., Zhang Q., McMichael J.F., Wyczalkowski M.A. (2013). Mutational landscape and significance across 12 major cancer types. Nature.

[4] Helleday T., Petermann E., Lundin C., Hodgson B., Sharma R.A. (2008). DNA repair pathways as targets for cancer therapy. Nat. Rev. Cancer.

[5] Syljuasen R.G. (2007). Checkpoint adaptation in human cells. Oncogene.

[6] Kubara P.M., Kerneis-Golsteyn S., Studeny A., Lanser B.B., Meijer L., Golsteyn R.M. (2012). Human cells enter mitosis with damaged DNA after treatment with pharmacological concentrations of genotoxic agents. Biochem. J..

[7] Swift L.H., Golsteyn R.M. (2014). Genotoxic anti-cancer agents and their relationship to DNA damage, mitosis, and checkpoint adaptation in proliferating cancer cells. Int. J. Mol. Sci..

[8] Swift L.H., Golsteyn R.M. (2016). Cytotoxic amounts of cisplatin induce either checkpoint adaptation or apoptosis in a concentration-dependent manner in cancer cells. Biol. Cell.

[9] Swift L.H., Golsteyn R.M. (2016). The relationship between checkpoint adaptation and mitotic catastrophe in genomic changes in cancer cells. Genome Stability.

[10] Tkacz-Stachowska K., Lund-Andersen C., Velissarou A., Myklebust J.H., Stokke T., Syljuasen R.G. (2011). The amount of DNA damage needed to activate the radiation-induced G2 checkpoint varies between single cells. Radiother. Oncol..

[11] Lewis C.W., Golsteyn R.M. (2016). Cancer cells that survive checkpoint adaptation contain micronuclei that harbor damaged DNA. Cell Cycle.

[12] Terradas M., Martin M., Tusell L., Genesca A. (2010). Genetic activities in micronuclei: Is the DNA entrapped in micronuclei lost for the cell?. Mutat. Res..

[13] Fenech M., Kirsch-Volders M., Natarajan A.T., Surralles J., Crott J.W., Parry J., Norppa H., Eastmond D.A., Tucker J.D., Thomas P. (2011). Molecular mechanisms of micronucleus, nucleoplasmic bridge and nuclear bud formation in mammalian and human cells. Mutagenesis.

[14] Crasta K., Ganem N.J., Dagher R., Lantermann A.B., Ivanova E.V., Pan Y., Nezi L., Protopopov A., Chowdhury D., Pellman D. (2012). DNA breaks and chromosome pulverization from errors in mitosis. Nature.

[15] Stephens P.J., Greenman C.D., Fu B., Yang F., Bignell G.R., Mudie L.J., Pleasance E.D., Lau K.W., Beare D., Stebbings L.A. (2011). Massive genomic rearrangement acquired in a single catastrophic event during cancer development. Cell.

[16] Holland A.J., Cleveland D.W. (2012). Chromoanagenesis and cancer: Mechanisms and consequences of localized, complex chromosomal rearrangements. Nat. Med..

[17] Zhang C.Z., Spektor A., Cornils H., Francis J.M., Jackson E.K., Liu S., Meyerson M., Pellman D. (2015). Chromothripsis from DNA damage in micronuclei. Nature.

[18] Terradas M., Martin M., Genesca A. (2016). Impaired nuclear functions in micronuclei results in genome instability and chromothripsis. Arch. Toxicol..

[19] Fenech M. (2006). Cytokinesis-block micronucleus assay evolves into a “cytome” assay of chromosomal instability, mitotic dysfunction and cell death. Mutat. Res..

[20] Storchova Z., Kloosterman W.P. (2016). The genomic characteristics and cellular origin of chromothripsis. Curr. Opin. Cell Biol..

[21] Rode A., Maass K.K., Willmund K.V., Lichter P., Ernst A. (2015). Chromothripsis in cancer cells: An update. Int. J. Cancer.

[22] Kastan M.B., Bartek J. (2004). Cell-cycle checkpoints and cancer. Nature.

[23] Bartek J., Lukas J. (2007). DNA damage checkpoints: From initiation to recovery or adaptation. Curr. Opin. Cell Biol..

[24] Rausch T., Jones D.T., Zapatka M., Stutz A.M., Zichner T., Weischenfeldt J., Jager N., Remke M., Shih D., Northcott P.A. (2012). Genome sequencing of pediatric medulloblastoma links catastrophic DNA rearrangements with TP53 mutations. Cell.

[25] Soto M., Raaijmakers J.A., Bakker B., Spierings D.C.J., Lansdorp P.M., Foijer F., Medema R.H. (2017). P53 prohibits propagation of chromosome segregation errors that produce structural aneuploidies. Cell Rep..

[26] Paull T.T., Rogakou E.P., Yamazaki V., Kirchgessner C.U., Gellert M., Bonner W.M. (2000). A critical role for histone H2AX in recruitment of repair factors to nuclear foci after DNA damage. Curr. Biol..

[27] Dalal S.N., Schweitzer C.M., Gan J., DeCaprio J.A. (1999). Cytoplasmic localization of human cdc25C during interphase requires an intact 14-3-3 binding site. Mol. Cell. Biol..

[28] Rothblum-Oviatt C.J., Ryan C.E., Piwnica-Worms H. (2001). 14-3-3 binding regulates catalytic activity of human wee1 kinase. Cell Growth Differ..

[29] Jazayeri A., Falck J., Lukas C., Bartek J., Smith G.C., Lukas J., Jackson S.P. (2006). ATM- and cell cycle-dependent regulation of ATR in response to DNA double-strand breaks. Nat. Cell Biol..

[30] Syljuåsen R.G., Jensen S., Bartek J., Lukas J. (2006). Adaptation to the ionizing radiation-induced G2 checkpoint occurs in human cells and depends on checkpoint kinase 1 and polo-like kinase 1 kinases. Cancer Res..

[31] Zhang Y.W., Otterness D.M., Chiang G.G., Xie W., Liu Y.C., Mercurio F., Abraham R.T. (2005). Genotoxic stress targets human Chk1 for degradation by the ubiquitin-proteasome pathway. Mol. Cell.

[32] Solyom S., Pylkas K., Winqvist R. (2010). Screening for large genomic rearrangements of the BRIP1 and CHK1 genes in finnish breast cancer families. Fam. Cancer.

[33] Cahuzac N., Studeny A., Marshall K., Versteege I., Wetenhall K., Pfeiffer B., Leonce S., Hickman J.A., Pierre A., Golsteyn R.M. (2010). An unusual DNA binding compound, S23906, induces mitotic catastrophe in cultured human cells. Cancer Lett..

[34] Warmerdam D.O., Brinkman E.K., Marteijn J.A., Medema R.H., Kanaar R., Smits V.A. (2013). UV-induced G2 checkpoint depends on p38 MAPK and minimal activation of ATR-Chk1 pathway. J. Cell Sci..

[35] Demarcq C., Bunch R.T., Creswell D., Eastman A. (1994). The role of cell cycle progression in cisplatin-induced apoptosis in Chinese hamster ovary cells. Cell Growth Differ..

[36] Toczyski D.P., Galgoczy D.J., Hartwell L.H. (1997). CDC5 and CKII control adaptation to the yeast DNA damage checkpoint. Cell.

[37] Clifford B., Beljin M., Stark G.R., Taylor W.R. (2003). G2 arrest in response to topoisomerase II inhibitors: The role of p53. Cancer Res..

[38] Hall E.J., Giaccia A.J. (2006). Radiobiology for the Radiologist.

[39] Brown J.M., Attardi L.D. (2005). The role of apoptosis in cancer development and treatment response. Nat. Rev. Cancer.

[40] Sandell L.L., Zakian V.A. (1993). Loss of a yeast telomere: Arrest, recovery, and chromosome loss. Cell.

[41] Lee S.E., Moore J.K., Holmes A., Umezu K., Kolodner R.D., Haber J.E. (1998). Saccharomyces Ku70, Mre11/Rad50 and RPA proteins regulate adaptation to G2/M arrest after DNA damage. Cell.

[42] Dewey W.C., Ling C.C., Meyn R.E. (1995). Radiation-induced apoptosis: Relevance to radiotherapy. Int. J. Radiat. Oncol. Biol. Phys..

[43] Kisurina-Evgenieva O.P., Sutiagina O.I., Onishchenko G.E. (2016). Biogenesis of micronuclei. Biochemistry.

[44] Hoffelder D.R., Luo L., Burke N.A., Watkins S.C., Gollin S.M., Saunders W.S. (2004). Resolution of anaphase bridges in cancer cells. Chromosoma.

[45] Terradas M., Martin M., Tusell L., Genesca A. (2009). DNA lesions sequestered in micronuclei induce a local defective-damage response. DNA Repair.

[46] Xu B., Sun Z., Liu Z., Guo H., Liu Q., Jiang H., Zou Y., Gong Y., Tischfield J.A., Shao C. (2011). Replication stress induces micronuclei comprising of aggregated DNA double-strand breaks. PLoS ONE.

[47] Terradas M., Martin M., Hernandez L., Tusell L., Genesca A. (2012). Nuclear envelope defects impede a proper response to micronuclear DNA lesions. Mutat. Res..

[48] Lemaitre J.M., Geraud G., Mechali M. (1998). Dynamics of the genome during early xenopus laevis development: Karyomeres as independent units of replication. J. Cell Biol..

[49] Geraud G., Laquerriere F., Masson C., Arnoult J., Labidi B., Hernandezverdun D. (1989). 3-dimensional organization of micronuclei induced by colchicine in PtK1 cells. Exp. Cell Res..

[50] Fenech M. (1993). The cytokinesis-block micronucleus technique and its application to genotoxicity studies in human-populations. Environ. Health Perspect..

[51] Fenech M. (2007). Cytokinesis-block micronucleus cytome assay. Nat. Protoc..

[52] Hermine T., Jones N.J., Parry J.M. (1997). Comparative induction of micronuclei in repair-deficient and -proficient chinese hamster cell lines following clastogen or aneugen exposures. Mutat. Res..

[53] Kisurina-Evgen’eva O.P., Bryantseva S.A., Shtil’ A.A., Onishchenko G.E. (2006). Antitubulin agents can initiate different apoptotic pathways. Biophysics.

[54] Heit R., Rattner J.B., Chan G.K., Hendzel M.J. (2009). G2 histone methylation is required for the proper segregation of chromosomes. J. Cell Sci..

[55] Luzhna L., Kathiria P., Kovalchuk O. (2013). Micronuclei in genotoxicity assessment: From genetics to epigenetics and beyond. Front. Genet..

[56] Shibata T., Shibamoto Y., Sasai K., Oya N., Murata R., Takagi T., Hiraoka M., Takahashi M., Abe M. (1996). Tirapazamine: Hypoxic cytotoxicity and interaction with radiation as assessed by the micronucleus assay. Br. J. Cancer Suppl..

[57] Snyder R.D., Diehl M.S. (2005). Hypoxia-induced micronucleus formation in mice. Drug Chem. Toxicol..

[58] Kelland L. (2007). The resurgence of platinum-based cancer chemotherapy. Nat. Rev. Cancer.

[59] Krishnaja A.P., Sharma N.K. (2004). Transmission of gamma-ray-induced unstable chromosomal aberrations through successive mitotic divisions in human lymphocytes in vitro. Mutagenesis.

[60] Janssen A., van der Burg M., Szuhai K., Kops G.J., Medema R.H. (2011). Chromosome segregation errors as a cause of DNA damage and structural chromosome aberrations. Science.

[61] Gieni R.S., Chan G.K., Hendzel M.J. (2008). Epigenetics regulate centromere formation and kinetochore function. J. Cell. Biochem..

[62] Bakhoum S.F., Compton D.A. (2012). Kinetochores and disease: Keeping microtubule dynamics in check!. Curr. Opin. Cell Biol..

[63] Gelot C., Magdalou I., Lopez B.S. (2015). Replication stress in mammalian cells and its consequences for mitosis. Genes.

[64] Cimini D., Howell B., Maddox P., Khodjakov A., Degrassi F., Salmon E.D. (2001). Merotelic kinetochore orientation is a major mechanism of aneuploidy in mitotic mammalian tissue cells. J. Cell Biol..

[65] Cimini D., Fioravanti D., Salmon E.D., Degrassi F. (2002). Merotelic kinetochore orientation versus chromosome mono-orientation in the origin of lagging chromosomes in human primary cells. J. Cell Sci..

[66] Gregan J., Polakova S., Zhang L., Tolic-Norrelykke I.M., Cimini D. (2011). Merotelic kinetochore attachment: Causes and effects. Trends Cell Biol..

[67] Cimini D., Wan X., Hirel C.B., Salmon E.D. (2006). Aurora kinase promotes turnover of kinetochore microtubules to reduce chromosome segregation errors. Curr. Biol..

[68] Bakhoum S.F., Thompson S.L., Manning A.L., Compton D.A. (2009). Genome stability is ensured by temporal control of kinetochore-microtubule dynamics. Nat. Cell Biol..

[69] Wang H.B., Peng B., Pandita R.K., Engler D.A., Matsunami R.K., Xu X.Z., Hegde P.M., Butler B.E., Pandita T.K., Mitra S. (2017). Aurora kinase B dependent phosphorylation of 53BP1 is required for resolving merotelic kinetochore-microtubule attachment errors during mitosis. Oncotarget.

[70] Shimizu N. (2011). Molecular mechanisms of the origin of micronuclei from extrachromosomal elements. Mutagenesis.

[71] Okamoto A., Utani K., Shimizu N. (2012). DNA replication occurs in all lamina positive micronuclei, but never in lamina negative micronuclei. Mutagenesis.

[72] Shimizu N., Shimura T., Tanaka T. (2000). Selective elimination of acentric double minutes from cancer cells through the extrusion of micronuclei. Mutat. Res..

[73] Shimizu N., Misaka N., Utani K. (2007). Nonselective DNA damage induced by a replication inhibitor results in the selective elimination of extrachromosomal double minutes from human cancer cells. Genes Chromosom. Cancer.

[74] Utani K., Okamoto A., Shimizu N. (2011). Generation of micronuclei during interphase by coupling between cytoplasmic membrane blebbing and nuclear budding. PLoS ONE.

[75] Medvedeva N.G., Panyutin I.V., Panyutin I.G., Neumann R.D. (2007). Phosphorylation of histone H2AX in radiation-induced micronuclei. Radiat. Res..

[76] Anderson D.J., Hetzer M.W. (2008). Reshaping of the endoplasmic reticulum limits the rate for nuclear envelope formation. J. Cell Biol..

[77] Lu L., Ladinsky M.S., Kirchhausen T. (2011). Formation of the postmitotic nuclear envelope from extended er cisternae precedes nuclear pore assembly. J. Cell Biol..

[78] Burke B., Ellenberg J. (2002). Remodelling the walls of the nucleus. Nat. Rev. Mol. Cell Biol..

[79] Gant T.M., Wilson K.L. (1997). Nuclear assembly. Annu. Rev. Cell Dev. Biol..

[80] Obe G., Beek B., Vaidya V.G. (1975). The human leukocyte test system. III. Premature chromosome condensation from chemically and X-ray induced micronuclei. Mutat. Res..

[81] Hatch E.M., Fischer A.H., Deerinck T.J., Hetzer M.W. (2013). Catastrophic nuclear envelope collapse in cancer cell micronuclei. Cell.

[82] Spann T.P., Moir R.D., Goldman A.E., Stick R., Goldman R.D. (1997). Disruption of nuclear lamin organization alters the distribution of replication factors and inhibits DNA synthesis. J. Cell Biol..

[83] Spann T.P., Goldman A.E., Wang C., Huang S., Goldman R.D. (2002). Alteration of nuclear lamin organization inhibits rna polymerase II—Dependent transcription. J. Cell Biol..

[84] Tang C.W., Maya-Mendoza A., Martin C., Zeng K., Chen S., Feret D., Wilson S.A., Jackson D.A. (2008). The integrity of a lamin-B1-dependent nucleoskeleton is a fundamental determinant of RNA synthesis in human cells. J. Cell Sci..

[85] Meyerson M., Pellman D. (2011). Cancer genomes evolve by pulverizing single chromosomes. Cell.

[86] Apraiz A., Boyano M.D., Asumendi A. (2011). Cell-centric view of apoptosis and apoptotic cell death-inducing antitumoral strategies. Cancers.

[87] Chang B.D., Broude E.V., Dokmanovic M., Zhu H., Ruth A., Xuan Y., Kandel E.S., Lausch E., Christov K., Roninson I.B. (1999). A senescence-like phenotype distinguishes tumor cells that undergo terminal proliferation arrest after exposure to anticancer agents. Cancer Res..

[88] Mak J.P., Man W.Y., Chow J.P., Ma H.T., Poon R.Y. (2015). Pharmacological inactivation of Chk1 and Wee1 induces mitotic catastrophe in nasopharyngeal carcinoma cells. Oncotarget.

[89] Petsalaki E., Dandoulaki M., Morrice N., Zachos G. (2014). Chk1 protects against chromatin bridges by constitutively phosphorylating BLM serine 502 to inhibit BLM degradation. J. Cell Sci..

[90] Magrangeas F., Avet-Loiseau H., Munshi N.C., Minvielle S. (2011). Chromothripsis identifies a rare and aggressive entity among newly diagnosed multiple myeloma patients. Blood.

[91] Molenaar J.J., Koster J., Zwijnenburg D.A., van Sluis P., Valentijn L.J., van der Ploeg I., Hamdi M., van Nes J., Westerman B.A., van Arkel J. (2012). Sequencing of neuroblastoma identifies chromothripsis and defects in neuritogenesis genes. Nature.

[92] Hirsch D., Kemmerling R., Davis S., Camps J., Meltzer P.S., Ried T., Gaiser T. (2013). Chromothripsis and focal copy number alterations determine poor outcome in malignant melanoma. Cancer Res..

[93] Garsed D.W., Marshall O.J., Corbin V.D., Hsu A., Di Stefano L., Schroder J., Li J., Feng Z.P., Kim B.W., Kowarsky M. (2014). The architecture and evolution of cancer neochromosomes. Cancer Cell.

[94] Carroll S.M., DeRose M.L., Gaudray P., Moore C.M., Needham-Vandevanter D.R., Von Hoff D.D., Wahl G.M. (1988). Double minute chromosomes can be produced from precursors derived from a chromosomal deletion. Mol. Cell. Biol..

[95] Zhang C.Z., Leibowitz M.L., Pellman D. (2013). Chromothripsis and beyond: Rapid genome evolution from complex chromosomal rearrangements. Genes Dev..

[96] Liu P., Erez A., Nagamani S.C., Dhar S.U., Kolodziejska K.E., Dharmadhikari A.V., Cooper M.L., Wiszniewska J., Zhang F., Withers M.A. (2011). Chromosome catastrophes involve replication mechanisms generating complex genomic rearrangements. Cell.

[97] Liu P., Carvalho C.M., Hastings P.J., Lupski J.R. (2012). Mechanisms for recurrent and complex human genomic rearrangements. Curr. Opin. Genet. Dev..

[98] Kinsella M., Patel A., Bafna V. (2014). The elusive evidence for chromothripsis. Nucleic Acids Res..

[99] Zeng X., Sigoillot F., Gaur S., Choi S., Pfaff K.L., Oh D.C., Hathaway N., Dimova N., Cuny G.D., King R.W. (2010). Pharmacologic inhibition of the anaphase-promoting complex induces a spindle checkpoint-dependent mitotic arrest in the absence of spindle damage. Cancer Cell.

[100] Sackton K.L., Dimova N., Zeng X., Tian W., Zhang M., Sackton T.B., Meaders J., Pfaff K.L., Sigoillot F., Yu H. (2014). Synergistic blockade of mitotic exit by two chemical inhibitors of the APC/C. Nature.

[101] Visconti R., Monica R.D., Palazzo L., D’Alessio F., Raia M., Improta S., Villa M.R., Del Vecchio L., Grieco D. (2015). The Fcp1-Wee1-Cdk1 axis affects spindle assembly checkpoint robustness and sensitivity to antimicrotubule cancer drugs. Cell Death Differ..

[102] Hanahan D., Weinberg R.A. (2011). Hallmarks of cancer: The next generation. Cell.

[103] Curtin N.J. (2012). DNA repair dysregulation from cancer driver to therapeutic target. Nat. Rev. Cancer.

[104] Visconti R., Monica R.D., Grieco D. (2016). Cell cycle checkpoint in cancer: A therapeutically targetable double-edged sword. J. Exp. Clin. Cancer Res..

